# Treatment of refractory cutaneous ulcers with mixed sheets consisting of peripheral blood mononuclear cells and fibroblasts

**DOI:** 10.1038/srep28538

**Published:** 2016-06-22

**Authors:** Koji Ueno, Yuriko Takeuchi, Makoto Samura, Yuya Tanaka, Tamami Nakamura, Arata Nishimoto, Tomoaki Murata, Tohru Hosoyama, Kimikazu Hamano

**Affiliations:** 1 Center for Regenerative Medicine, Yamaguchi University Graduate School of Medicine, Ube, Japan; 2 Department of Surgery and Clinical Sciences, Yamaguchi University Graduate School of Medicine, Ube, Japan; 3 Institute of Laboratory Animals, Yamaguchi University, Ube, Japan

## Abstract

The purpose of this study was to confirm the therapeutic effects of mixed sheets consisting of peripheral blood mononuclear cells (PBMNCs) and fibroblasts on cutaneous skin ulcers. Vascular endothelial growth factor (VEGF) secretion in mixed cell sheets was much higher than in PBMNCs and fibroblasts. Concerning the mechanism, transforming growth factor beta 1 and platelet-derived growth factor BB secreted from PBMNCs enhanced VEGF production in fibroblasts. In wounds created on the backs of diabetic mice, the therapeutic effect of mixed cell sheets was similar to that of daily treatment with trafermin, a recombinant human basic fibroblast growth factor. Although abnormal granulation tissue and inflammatory cell infiltration were observed in trafermin-treated wounds, the transplantation of mixed cell sheets resulted in the natural anatomy of subcutaneous tissues. The expression patterns of identical wound-healing factors in wounds were different between mixed sheet-transfected and trafermin-treated animals. Because mixed cell sheets transplanted into full-thickness skin defects were eliminated in hosts by day 21 in syngeneic transplantation models, allogeneic transplantation was performed using mice with different genetic backgrounds. The wound-healing rates were similar between the mixed cell sheet and trafermin groups. Our data indicated that mixed cell sheets represent a promising therapeutic material for cutaneous ulcers.

More than 200 million people are affected by peripheral arterial disease globally^1^. Although revascularization via endovascular treatment and bypass surgery is the best treatment for critical limb ischemia (CLI), difficulties can be experienced, and some patients are not suitable for revascularization surgery^2^. Even if surgical treatment is successful, if a local circulatory disorder remains, the cutaneous ulcer could be refractory. Regarding patients unsuitable for neovascularization surgery, clinical trials of bone marrow cell transplantation in patients with CLI have been conducted^3^. Although these trials revealed a certain therapeutic effect for neovascularization, it was noted that the survival rate of transplanted cells was extremely low in the ischemic tissue^4^,^5^ because transplanted cells are attacked by reactive oxygen species accumulated in ischemic areas, and most cells are destroyed via apoptosis and necrosis without exerting an angiogenic effect^6^,^7^.

To overcome this challenge, we previously reported a method to enhance the survival rate and growth factor production of implanted cells called “hypoxic preconditioning.” This method increased neovascularization in rats with hindlimb ischemia using bone marrow cells^8^. Hypoxic preconditioning increased the vascular endothelial growth factor (VEGF) production, cell survival rate, and cellular adhesive ability in rodent bone marrow cells, resulting in a higher therapeutic efficacy after transplantation in ischemic hindlimbs^9^. To reduce invasiveness, we utilized peripheral blood mononuclear cells (PBMNCs) instead of bone marrow cells and further examined the therapeutic effects of preconditioned PBMNCs on ischemic hindlimbs. Similar to the findings for bone marrow cells, hypoxic preconditioning enhanced oxidative stress resistance in PBMNCs. Hypoxically pretreated PBMNCs improved microvessel density and limb blood flow in both mouse and rabbit hindlimb ischemia models^10^,^11^.

To improve the retention of cells in grafted regions, cell sheet technology has been developed^12^, and its therapeutic availability has been demonstrated in a variety of disease models^13^,^14^,^15^,^16^. Because the poor retention of graft cells or artificial skins in wounded areas is one of the primary factors to induce insufficient repair of refractory ulcers, we believed that cell sheet technology may be a powerful therapeutic tool for refractory cutaneous ulcers caused by critical limb ischemia. In this study, we developed novel cell sheets comprising PBMNCs mixed with fibroblasts and examined their efficacy against cutaneous ulcer using diabetic mouse models. Mixed cell sheets have the advantages of sustained growth factor secretion. In fact, the production of VEGF in mixed cell sheets was significantly higher than in fibroblast sheets, suggesting that mixed cell sheets possess a prominent angiogenic activity. The therapeutic effect of cell sheet delivery in cutaneous ulcers was similar or better than that of trafermin, a recombinant human basic fibroblast growth factor, and similar therapeutic effects were also observed in allogeneic transplantation models. Taken together, mixed cell sheets represent a useful new tool to treat cutaneous ulcers.

## Results

### Enhanced secretion of VEGF in mixed cell sheets

**Figure 1:**
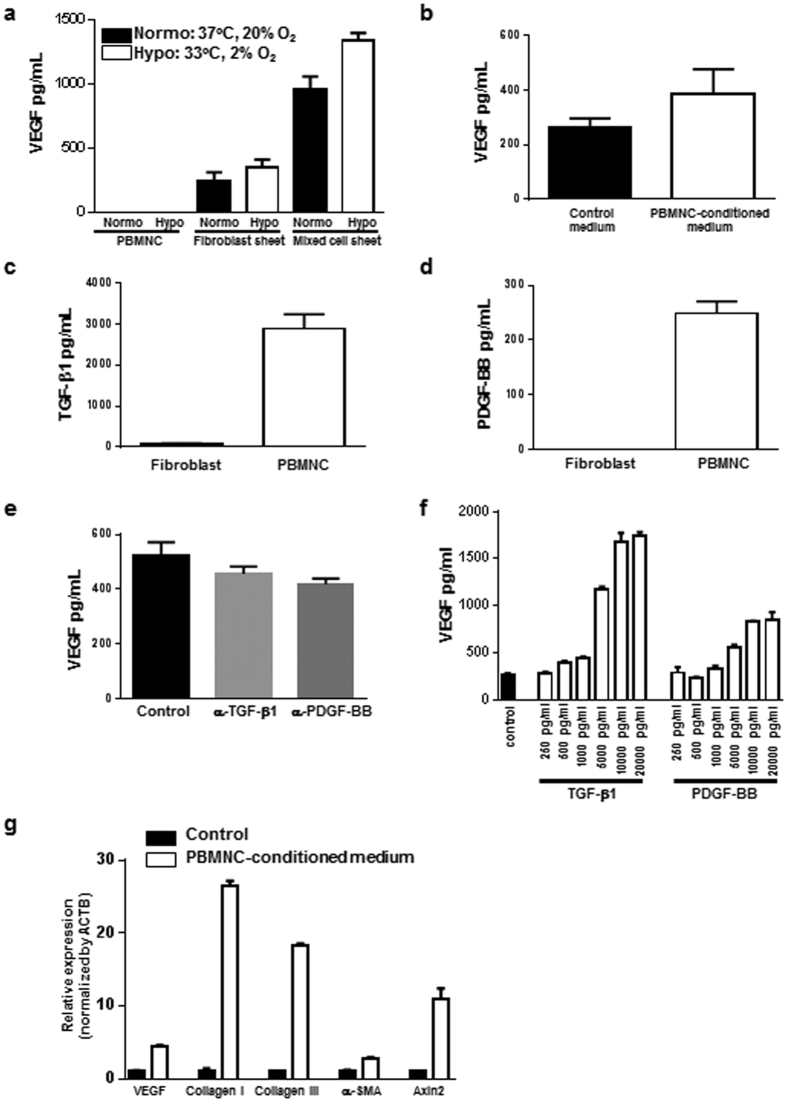
Synergetic effect of mixed sheets consisting of PBMNCs and fibroblasts. (**a**) Comparison of VEGF secretion. The VEGF concentration in the culture medium was measured in PBMNCs, fibroblast sheets, and mixed cell sheets for 3 days. Normoxic condition: 37 °C, 20% O_2_ for 3 days. Hypoxic condition: 37 °C, 20% O_2_ for 2 days followed by 33 °C, 2% O_2_ for 1 day. (**b**) Secretion from PBMNCs increased VEGF production by fibroblasts. Fibroblasts were cultured for 48 h with or without the PBMNC-conditioned medium, and the VEGF concentration in the supernatant was analyzed by ELISA. (**c**) TGF-β1 concentration in fibroblasts and PBMNC culture medium at 48 h. (**d**) PDGF-BB concentration in fibroblasts and PBMNC culture medium at 48 h. (**e**) Neutralizing antibody against TGF-β1 and PDGF-BB inhibited VEGF production in fibroblasts. The PBMNC-conditioned medium was co-cultured with a neutralizing antibody against TGF-β1 or PDGF-BB, and the PBMNC-conditioned medium was added to fibroblasts. After 48 h, the VEGF concentration was measured by ELISA. (**f**) TGF-β1 and PDGF-BB recombinant proteins elevated VEGF production by fibroblasts. (**g**) The PBMNC-conditioned medium increased the expression levels of VEGF, collagen I, collagen III, α-SMA, and Axin2 mRNA. Fibroblasts were cultured with the PBMNC-conditioned or control medium for 48 h. The mRNA expression levels were determined using real-time PCR. ACTB was used as an endogenous control. The expression levels were compared with that in control medium, which is presented as 1.

To assess the effect of mixed cell sheets, the concentration of VEGF in the supernatant was measured using enzyme-linked immunosorbent assay (ELISA) at 3 days after incubation in UpCell^®^ 24 multi-well plates. The VEGF concentrations in mixed cell sheets in both normoxic and hypoxic conditions were more than threefold higher than those in fibroblast sheets. In contrast, PBMNCs did not secrete VEGF regardless of the culture conditions (Fig. 1a). To uncover the mechanism of the upregulation of VEGF in mixed cell sheets, fibroblasts were cultured in a PBMNC-conditioned medium. Fibroblasts displayed increased VEGF secretion in response to the PBMNC-conditioned medium (Fig. 1b). Conversely, the fibroblast-conditioned medium did not induce VEGF production in PBMNCs (data not shown). To identify specific soluble factors secreted from PBMNCs, we explored cytokine and growth factors using the supernatant of PBMNCs and fibroblasts. In this assay, we found that transforming growth factor beta 1 (TGF-β1) and platelet-derived growth factor BB (PDGF-BB) were secreted specifically by PBMNCs but not by fibroblasts (Fig. 1c,d). To clarify whether these factors from PBMNCs are soluble enhancers for VEGF expression in fibroblasts, the PBMNC-conditioned medium was neutralized with antibodies against TGF-β1 and PDGF-BB prior to incubation with fibroblasts. VEGF secretion was significantly decreased in fibroblasts cultivated in a neutralized PBMNC-conditioned medium (Fig. 1e). In contrast, recombinant TGF-β1 and PDGF-BB proteins increased the concentration of VEGF in fibroblasts in a concentration-dependent manner (Fig. 1f). Higher levels of IL-16, CCL5, and IL-6 were detected in fibroblast-conditioned media and in media conditioned with PBMNCs and fibroblasts compared with a PBMNC-conditioned medium using cytokine array analysis (Supplementary Figure S1a). However, recombinant IL-16, CCL5, and IL-6 proteins did not increase the secretion of VEGF by fibroblasts (Supplementary Figure S1b). To confirm whether soluble factors from PBMNCs activate wound healing-associated factors in fibroblasts, fibroblasts were cultured in the PBMNC-conditioned medium and then the gene expression of VEGF, collagen I, collagen III, α-SMA, and Axin2 was analyzed using quantitative polymerase chain reaction (qPCR). As a result, we found that genes with relevance to wound healing were upregulated in fibroblasts (Fig. 1g).

### Therapeutic effect of mixed cell sheets in a mouse cutaneous ulcer model

**Figure 2:**
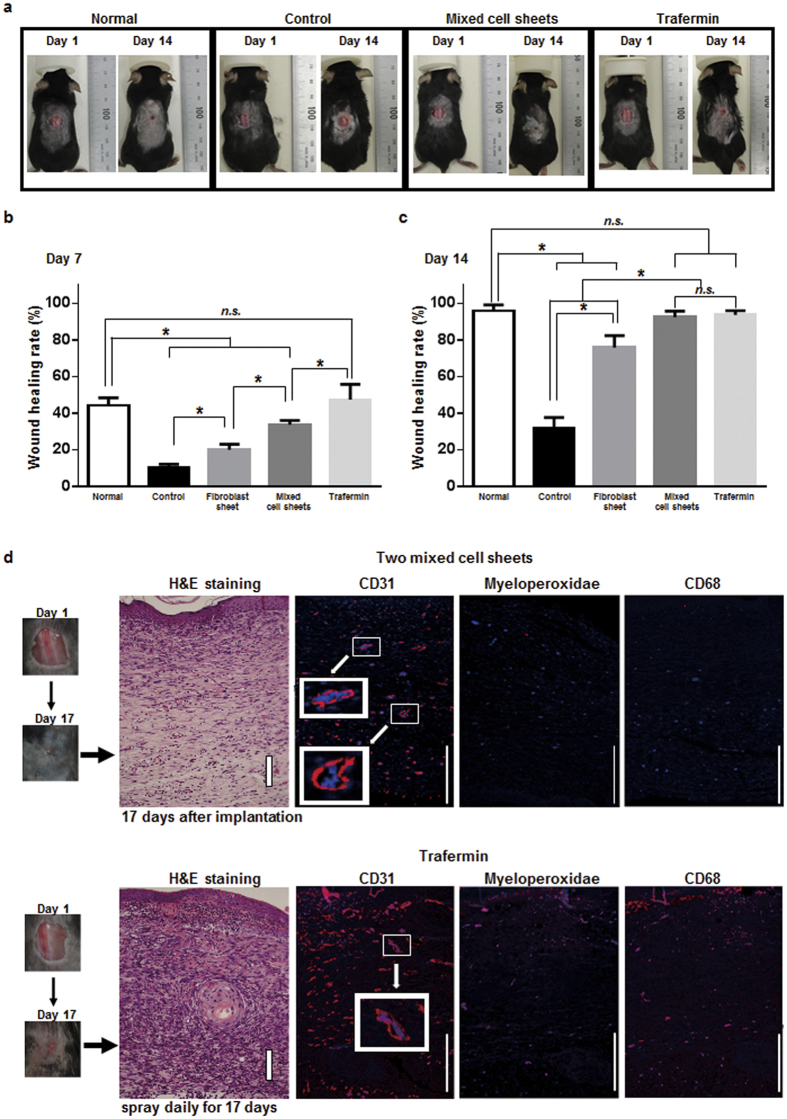
Effect on mixed cell sheets as therapeutic materials. (**a**) Wound healing after fibroblast sheet transplantation, mixed cell sheet transplantation, and trafermin treatment. The images show the full-thickness skin defects on the backs of diabetic mice on days 1 and 14. Normal shows normal and non-DM mice, which do not receive the experimental treatment. Control shows DM mice, which do not receive the experimental treatment. (**b**) The wound healing rate 7 days after treatment. Each group included six mice. (**c**) The wound healing rate 14 days after treatment. Each group included six mice. (**d**) Typical H&E, CD31, Myeloperoxidae and CD68 staining images 17 days after mixed cell sheet transplantation and trafermin treatment. CD31 is a marker endothelial cell. Myeloperoxidae is a marker of neutrophil. CD68 is a marker of macrophage. Bar shows 200 μm.

To evaluate mixed cell sheets as a therapeutic material for cutaneous ulcers, cell sheets originating from C57BL/6 mice were transplanted onto full-thickness skin defects of the backs in normal and syngeneic diabetes mellitus (DM) mice (Fig. 2a). At 7 days after the initiation of therapy, the wound healing rate was significantly higher in the mixed cell sheets group (mean, 33.79%) than in the control (10.51%) and fibroblast sheet groups (20.10%), but the rate was significantly lower than in the trafermin group (47.40%) (Fig. 2b). At 14 days, the wound healing rate of the mixed cell sheets group was equivalent to the trafermin group (92.57% vs. 93.61%) and significantly higher than in the control (31.76%) and fibroblast sheet groups (75.93%) (Fig. 2c). Wound healing rates in normal mice were 44.34% at 7 days and 95.98% at 14 days (Fig. 2b,c). Skin defects on the backs in DM mice were completely closed by 17 days after treatment in both the mixed cell sheets and trafermin groups (Fig. 2d). In histological analysis of the wounds, however, an obvious difference in tissue regeneration was found between the mixed cell sheets and trafermin groups. Although tissues from mice treated with the mixed cell sheets displayed a natural anatomy, inflammatory cells including neutrophils and macrophages were frequently observed in trafermin-treated tissues, indicating that abnormal tissue regeneration was induced in trafermin-treated mice. CD31 staining revealed microvessel-like structures in tissues treated with the mixed cell sheets.

### Analysis of the wound healing process in mixed cell sheet-treated mice

**Figure 3:**
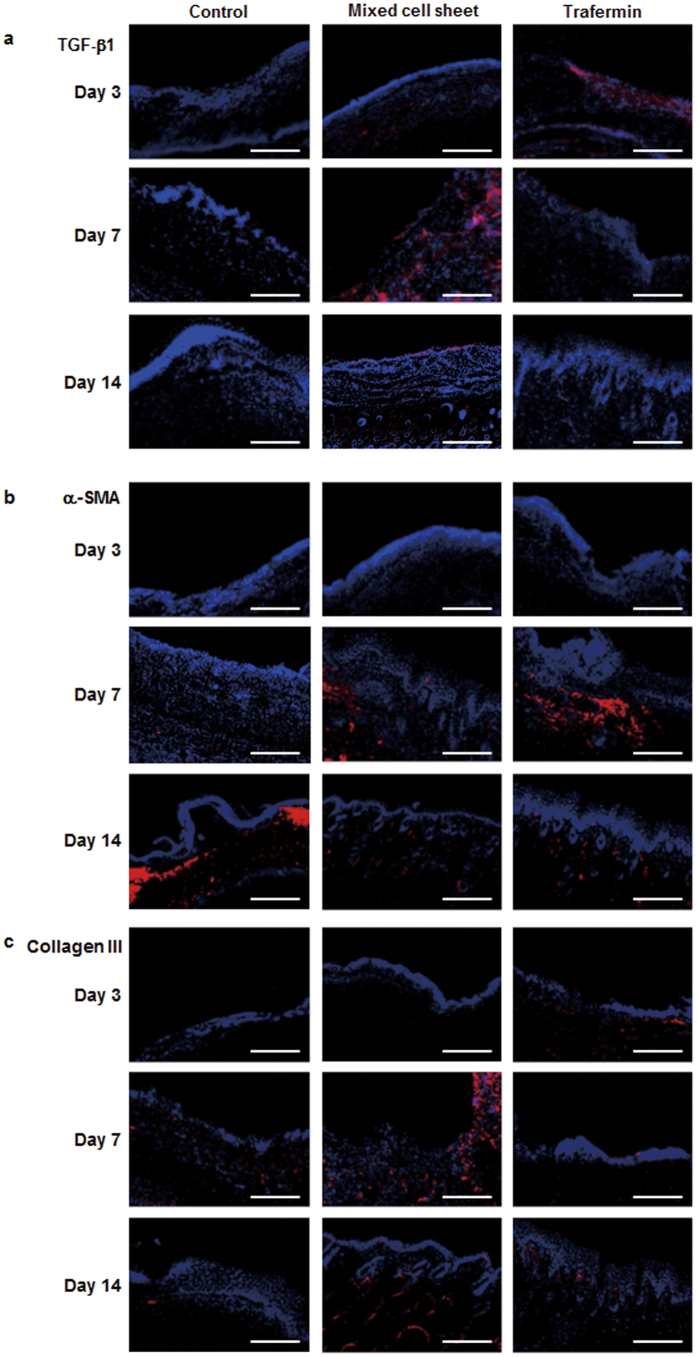
Immunostaining against markers of the wound healing process 3, 7, and 14 days after mixed cell sheet transplantation and trafermin treatment. (**a**) TGF-β1. (**b**) α-SMA. (**c**) Collagen III. Bar shows 200 μm.

To analyze the chronological changes of tissue regeneration in a wound after the transplantation of mixed cell sheets, the expression of TGF-β1, α-SMA, and collagen III in a wound was temporally monitored after the initiation of therapy (Fig. 3a–c). In trafermin-treated tissues, the strong expression of TGF-β1 and α-SMA was observed on days 3 and 7, respectively. In cell sheet-transplanted tissues, the strong expression of TGF-β1, α-SMA, and collagen III was observed on day 7. Concerning the control, TGF-β1 and α-SMA were strongly expressed on day 14. These results reveal differences in the healing process between the therapeutic approaches.

### Analysis of whether transplanted fibroblasts remain on the host

**Figure 4:**
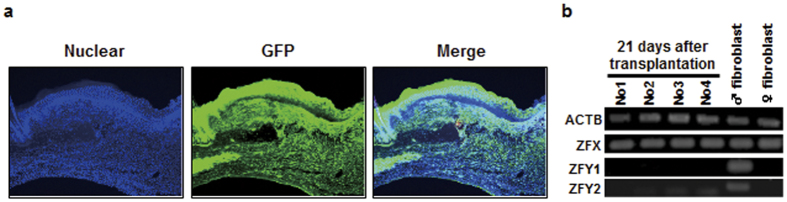
Persistence on mixed cell sheets in cutaneous tissues. (**a**) Mixed cell sheets did not expand in cutaneous tissues. Mixed cell sheets originating from wild-type male C57BL/6 mice were transplanted into full-thickness skin defects on the backs of male C57BL/6 GFP-transgenic mice. After 14 days, healing tissues were stained using the anti-GFP antibody. The left image shows DAPI staining of the nucleus. The middle image shows GFP staining. The right image is the merged image of DAPI and GFP staining. (**b**) Y-chromosome analysis for male-derived cell sheet transplantation using PCR. Mixed cell sheets derived from C57BL/6 male mice were transplanted into full-thickness skin defects on the backs of diabetic female C57BL/6 mice. After 21 days, genomic DNA was extracted from wounded tissues. The existence of ZFY1 and ZFY2 encoded on the Y-chromosome was analyzed using PCR. ACTB and ZFX encoded on the X-chromosome were used as experimental controls.

To examine whether fibroblasts in mixed cell sheets expand in host tissues, mixed cell sheets originating from wild-type mice were transplanted into full-thickness skin defects of the back in green fluorescent protein (GFP)-transgenic mice, and the histology of wounded tissues was analyzed at day 14 after transplantation (Fig. 4a). As a result, all cells in wounded areas expressed GFP, suggesting that fibroblasts in the cell sheets never expanded in the host after transplantation. To further confirm cell expansion from mixed cell sheets in the host, mixed cell sheets derived from wild-type male mice were transplanted into wild-type female mice with full-thickness skin defects of the back. Genomic DNA was extracted from the wounded tissue at day 21 after transplantation, and the expression of male-specific genes, namely ZFY1 and ZFY2, on the Y-chromosome in the tissue was assessed by PCR (Fig. 4b). Although the ZFX gene encoded on the X-chromosome was detected in all samples, both male-specific genes were undetectable in wounded female mice. This result indicates that transplanted cell sheets may be eliminated from the cutaneous tissue in the host.

### Allogeneic mixed cell sheets as therapeutic materials

**Figure 5:**
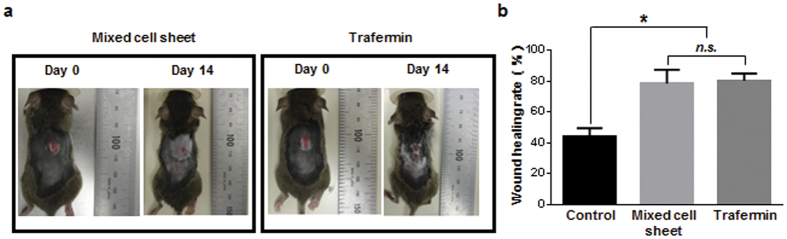
Therapeutic effect of allogeneic mixed cell sheets. (**a**) Wound healing effect of allogeneic mixed cell sheet transplantation and trafermin treatment. Allogeneic mixed cell sheets originating from C57BL/6 GFP-transgenic male mice were transplanted into full-thickness skin defects on the backs of diabetic male C3H mice. The images show the full-thickness skin defects on the backs of mice on days 1 and 14. (**b**) Wound healing rate 14 days after treatment. The control groups included five mice. The allogeneic mixed cell sheet group included nine mice. The trafermin treatment group included seven mice.

The elimination of transplanted sheets from host animals raised the possibility of the allogeneic transplantation of mixed cell sheets in consideration of future clinical applications. To evaluate this possibility, we performed allogeneic transplantation experiments using mice with different genetic backgrounds. Mixed cell sheets were prepared from GFP-expressing C57BL/6 mice and then transplanted into wounded C3H mice (Fig. 5a). At day 14 after treatment, the wound healing rate of sheet-implanted mice was significantly higher than that of non-sheet delivered mice (78.57% vs. 44.36%) and was equivalent to that of trafermin-treated mice (80.33%) (Fig. 5b).

Transplanted GFP-expressing cells were not detected in C3H host cells 23 days after allogenic mixed cell sheet transplantation (Supplementary Figure S2).

## Discussion

Our present data demonstrated that mixed cell sheets exhibited a higher VEGF production than sheets of either PBMNCs or fibroblasts. We also demonstrated that fibroblasts secrete lower levels of PDGF-BB and TGF-β1, and VEGF secretion was concentration-dependently upregulated in fibroblast cultures by the administration of TGF-β1 and PDGF-BB. Taken together, it is suspected as a possible mechanism that TGF-β1 and PDGF-BB secreted from PBMNCs are critical factors that induce VEGF production in fibroblasts. It has been reported that some factors secreted from PBMNCs transplanted into the ischemic hindlimb stimulates IL-1β expression in muscle tissues and that IL-1β induces neovascularization via an increase in VEGF expression in the ischemic hindlimb^17^. This report supports our data that soluble factors from PBMNCs enhanced VEGF production in fibroblasts.

Although the wound healing rate in the cell sheet implantation group was equivalent to that in the trafermin administration group at day 14 after treatment, abnormal granulation tissue and inflammatory cell infiltration were observed in the wounded tissue in trafermin-treated mice in contrast to the natural healing observed in sheet-implanted tissue. This result suggests that mixed cell sheet implantation is superior to existing medication regarding pathological outcomes.

TGF-β1, α-SMA, and collagen III are important factors for wound repair, and they are generally used as typical markers of the wound healing process. TGF-β1 induces the differentiation of fibroblasts to α-SMA-expressing myofibroblasts, and myofibroblasts then secrete a wide variety of matrices such as collagen III^18^,^19^,^20^,^21^. In this study, we analyzed the expression profile of wound healing-associated factors in wounded tissues after treatment with cell sheets or trafermin. The expression patterns of TGF-β1, α-SMA, and collagen III were different between the cell sheet and trafermin groups. TGF-β1 expression was observed on day 3 in the trafermin group and on day 7 in the mixed cell sheets group. Collagen III expression was observed in the cell sheet transplantation group on day 7. These data support the findings that the wound healing rate in the mixed cell sheet transplantation group was lower than that in the trafermin group on day 7 and that wounds treated with transplanted mixed cell sheets rapidly shrank after day 7. Figure 2 shows that the average wound healing period was 17 following mixed cell sheet treatment and 20 days following fibroblast sheet treatment. Figure 1g shows that PBMNC-conditioned medium increased the expression levels of VEGF, collagen I, and collagen III mRNAs in fibroblasts. These findings suggest that mixed cell sheets secreted more collagen I and III than fibroblast sheets. This may explain the advanced wound healing.

The survival rate of skeletal myoblast sheets transplanted into infarcted hearts was slightly less than 20% in a rat model on day 28, and oral mucosal epithelial cell sheets transplanted into esophageal ulceration were observed on day 8 in a canine model^22^,^23^.

On the contrary, mixed cell sheets transplanted into the wounded cutaneous tissue of DM mice were eliminated in the host by day 21. Unlike skeletal myoblast sheets transplanted into infarcted hearts, mixed cell sheets were transplanted into skin defected tissues in which cutaneous tissues were greatly repaired as a cutaneous ulcer model. Therefore, mixed cell sheets may be excluded during tissue repair. CD11b-positive cells from PBMNCs were incorporated into the mixed cell sheets (Supplementary Figure S3), but these may not have included lymphocytes as these cells are non-adhering.

In the present study, we demonstrated the possible autologous transplantation of mixed cell sheets using syngeneic mouse models. However, autologous transplantation requires more time and cost to prepare cell sheets, and it is needless to say that allogeneic transplantation is a much better therapeutic approach in clinical settings. To examine further applications of mixed cell sheets, we also performed allogeneic transplantation of cell sheets into mice of different genetic backgrounds and genders. In both experiments, the wound healing rate in the sheet-delivered group was much higher than that in the non-sheet–delivered group but was similar to that in the trafermin-treated group. Importantly, delivered cell sheets were not incorporated into host animals, suggesting that mixed cell sheets were rapidly eliminated from the host tissue during wound recovery. Taken together, our results indicate the possibility that mixed cell sheets are useful for allogeneic transplantation. Because it takes more time to prepare autologous cell sheets, the transplantation of allogeneic cell sheets has an advantage of shortening the sheet preparation time when considering clinical applications.

In conclusion, this is the first report to illustrate the therapeutic applicability of mixed cell sheets consisting of PBMNCs and fibroblasts for treating cutaneous skin ulcers. Two different types of cells in the mixed sheet synergistically affected each other to enhance therapeutic properties such as VEGF production, resulting in a higher therapeutic efficacy in skin ulcers. Although additional detailed studies are needed, we believe that mixed cell sheets will become powerful therapeutic materials for treating refractory cutaneous skin ulcers in clinical settings.

## Materials and Methods

### Animals

Male/female C57BL/6 and male C3H mice were purchased from Japan SLC, Inc. (Shizuoka, Japan). Male GFP-transgenic mice (C57BL6/Tg14) were provided by Masaru Okabe (Genome Research Center, Osaka University, Osaka)^24^. All animal procedures were approved by the Institutional Animal Care and Use Committee of Yamaguchi University (#31-093). The methods were conducted in accordance with the approved guidelines.

### Isolation of PBMNCs and fibroblasts

PBMNCs were isolated from mouse peripheral blood using Lympholyte^®^-M (CedarLane Laboratories Ltd., Hornsby, Ontario, Canada) and cultured in RPMI-1640 (Thermo Fisher Scientific, Waltham, MA, USA) supplemented with 10% heat-inactivated fetal bovine serum (Thermo Fisher Scientific) and penicillin-streptomycin (Thermo Fisher Scientific). Fibroblasts were isolated from the tails of mice using collagenase (Wako, Osaka, Japan) and cultured in DMEM (Thermo Fisher Scientific) supplemented with 20% heat-inactivated fetal bovine serum, MEM non-essential amino acids solution (Life Technologies), and penicillin-streptomycin.

### Preparation of cell sheets

For mixed cell sheets, 1 ml of PBMNCs (2 × 10^6^ cell/ml) and 1 ml of fibroblasts (1.25 × 10^5^ cell/ml) were added to the same well in UpCell^®^ 24 multi-well plates (CellSeed Inc., Tokyo, Japan). For fibroblast sheets, 1 ml of fibroblasts (1.25 × 10^5^ cell/ml) and 1 ml of RPMI-1640 including supplements were added to the same well in UpCell^®^ 24 multi-well plates. For PBMNC cultures, 1 ml of PBMNCs (2 × 10^6^ cell/ml) and 1 ml of DMEM with supplements were added to the same well in UpCell^®^ 24 multi-well plates. For hypoxic preconditioning, cells were incubated for 2 days under normoxic conditions (37 °C in 20% O_2_ and 5% CO_2_) followed by 1 day under hypoxic conditions (33 °C in 2% O_2_ and 5% CO_2_). For normoxic preconditioning, cells were incubated for 3 days under normoxic conditions (37 °C in 20% O_2_ and 5% CO_2_).

### Cytokine assay

The concentrations of VEGF, TGF-β1, and PDGF-BB in the cell culture supernatant were measured using mouse ELISA kits (R&D Systems, Inc., Minneapolis, Minnesota, USA) according to the manufacturer’s instructions. Cytokines in conditioned media for PBMNCs, fibroblast, and co-culture of PBMNCs and fibroblast were measured using Proteome Profiler Mouse Cytokine Array Kit, Panel A (R&D Systems, Inc.)

### VEGF production and mRNA expression analysis in fibroblasts

PBMNCs (2 × 10^6^ cell/ml) were cultured for 48 h followed by centrifugation at 3000 rpm for 5 min, and the supernatant was used as a conditioned medium for PBMNCs. In the neutralizing antibody experiment, the conditioned medium was incubated for 90 min at 4 °C with anti-ATM antibody (38 μg/mL, ab78, Abcam, Cambridge, UK) as a control antibody, anti-TGF-β1 antibody (38 μg/mL, ab64715, Abcam), or anti-PDGF-BB antibody (33 μg/mL, AF-220-NA, R&D Systems, Inc.) and then added to fibroblasts. The cell culture supernatant was harvested after 48 h. The VEGF concentration was measured as described previously. In experiments with a recombinant protein, TGF-β1 (R&D Systems, Inc.), PDGF-BB (Sigma-Aldrich, St Louis, MO, USA), IL-16 (Sigma-Aldrich), CCL5 (Miltenyi Biotec, Bergisch Gladbach, Germany), and IL-6 (Miltenyi Biotec) recombinant proteins were added to fibroblasts. After 48 h of incubation, the VEGF concentration in the cell culture supernatant was analyzed as described previously. To analyze mRNA expression levels, fibroblasts were cultured with the PBMNC-conditioned or control medium for 48 h. Total RNA was extracted using an RNeasy Min Kit (Qiagen, Hilden, Germany), and the extracted total RNA was reverse-transcribed into single-stranded cDNA with a PrimeScript™ RT reagent Kit (Perfect Real Time) (TaKaRa Bio Inc, Kusatsu, Shiga, Japan). Real-time PCR was performed using first-strand cDNA with SYBR^®^ Select Master Mix (Thermo Fisher Scientific). The primers used were as follows: ACTB forward primer, 5′-gctcctcctgagcgcaag-3′; ACTB reverse primer, 5′-catctgctggaaggtggaca-3′; VEGF forward primer, 5′-ctttgttctgtctttctttggtctg-3′; VEGF reverse primer, 5′-atgcggatcaaacctcacc-3′; collagen I forward primer, 5′-aagaatggagatgatggggaag-3′; collagen I reverse primer, 5′-caatccacgagcaccctga-3′; collagen III forward primer, 5′-ctagactgccccaacccaga-3′; collagen III reverse primer, 5′-gccatcaggaagcacagga-3′; alpha-smooth muscle actin (α-SMA) forward primer, 5′-cgggagaaaatgacccaga-3′; α-SMA reverse primer, 5′-gtccagaggcatagagggacag-3′; Axin2 forward primer, 5′-ctgaaactggagctggaaagc-3′; and Axin2 reverse primer, 5′- acccctccttttcttcatcctc-3′. qPCR was performed using a StepOnePlus Real-Time PCR System (Thermo Fisher Scientific). The qPCR parameters for cycling were as follows: 50 °C for 2 min, 95 °C for 2 min, and 40 cycles of PCR at 95 °C for 3 s and 60 °C for 30 s. All reactions were performed in a 10-μl reaction volume in triplicate. The mRNA expression levels were determined using the 2^−ΔCT^ method.

### Cutaneous ulcer model and cell sheet transplantation

Mice were injected with streptozotocin (Sigma-Aldrich). Mice with blood glucose levels of more than 300 mg/dl were used for the animal experiments as DM mice and anesthetized via the inhalation of 1.5% isoflurane during the surgical procedure. To compare healing of skin defects in normal and DM nice, normal male mice were assigned to the experimental group. For mice with ulcers on the back, full-thickness skin defects of 8 mm in diameter were created using a surgical technique. For the experiments in which mixed cell sheets from male mice were transplanted in to females, cyclosporine (Novartis, Basel, Switzerland) was administrated on a weekly basis. Mixed cell sheets and fibroblast sheets cultured under hypoxic conditions were transplanted onto full-thickness skin defects. Trafermin was sprayed daily using Fiblast Spray (Kaken Pharmaceutical Co., Ltd., Tokyo, Japan). The area of the skin defect was measured using ImageJ software.

### Histological analysis

For hematoxylin and eosin (H&E) staining, after skin defects healed, cutaneous tissues were resected and fixed with neutral formalin (Wako). For fluorescent immunostaining, cutaneous tissues were resected and fixed by 4% paraformaldehyde for 2 h at 4 °C, followed by incubation in 15% sucrose for 4 h at 4 °C and then in 30% sucrose overnight at 4 °C. The tissues were embedded into OCT compound (Sakura Finetek Japan, Tokyo, Japan) and frozen at −80 °C. The antibodies used were as follows: anti-GFP pAb (598, MEDICAL & BIOLOGICAL LABORATORIES CO., LTD., Nagoya, Aichi, Japan), anti-TGF-β1 antibody (ab64715, Abcam), anti-collagen III antibody (ab7778, Abcam), anti-actin, α-SMA-Cy3™ antibody (C6198, Sigma-Aldrich), anti-CD31 antibody (ab28364, Abcam), anti-Myeloperoxidae antibody (ab9535), anti-CD68 antibody (ab31630), Biotin anti-mouse H-2K^k^ antibody (BioLegend, San Diego, California, USA), anti-CD11b antibody (16-0112, eBioscience, San Diego, California, USA), anti-mouse IgG (H+L) secondary antibody, Alexa Fluor^®^ 594 conjugate (A-11005, Thermo Fisher Scientific), anti-rabbit IgG (H+L) secondary antibody, Alexa Fluor^®^ 488 conjugate (A-11034, Thermo Fisher Scientific), anti-rat IgG (H+L) secondary antibody, Alexa Fluor^®^ 594 conjugate (A-11007, Thermo Fisher Scientific) and anti-rabbit IgG H&L (DyLight^®^ 550) (ab96884, Abcam), DyLight 594 Streptavidin (Vector Laboratories, Burlingame, California, USA).

### Y-chromosome analysis using PCR

Genomic DNA from wounded tissues in females at day 21 after the implantation of male mixed cell sheets was extracted using an AllPrep DNA/RNA Mini Kit (QIAGEN) and sonication (SLPe 40, BRANSON, Danbury, CT, USA). PCR was performed using genomic DNA with KOD FX (TOYOBO, Tokyo, Japan). The PCR parameters for cycling were as follows: 94 °C for 2 min, 40 cycles of PCR at 98 °C for 10 s, 62 °C for 30 s, and 68 °C for 30 s. All reactions were performed in a 10-μl volume containing 10 μg of genomic DNA, and 5 μl of the PCR product were analyzed using 2% Tris-acetate-EDTA (TAE) gel electrophoresis. The primers used were as follows: ACTB forward primer, 5′-ctggcagtcacccagagaca-3′; ACTB reverse primer, 5′-tggagtcaggcacttttggag-3′; ZFX forward primer, 5′-aaagctgatcctggagaagatga-3′; ZFX reverse primer, 5′-acccaaaagcaatcagactgaag-3′; ZFY1 forward primer, 5′-tcctttacagactgacaaagtgtga-3′; ZFY1 reverse primer, 5′-cacctaggcttctgaggctaaa-3′; ZFY2 forward primer, 5′-aatcaaggctgaggggtgtg-3′; and ZFY2 reverse primer, 5′-ccagagactggccccatagtt-3′.

### Statistical analysis

All statistical analyses were performed using GraphPad Prism 6 software (GraphPad Software, San Diego, CA, USA). A P-value of <0.05 was regarded as statistically significant.

## Additional Information

**How to cite this article**: Ueno, K. *et al.* Treatment of refractory cutaneous ulcers with mixed sheets consisting of peripheral blood mononuclear cells and fibroblasts. *Sci. Rep.* **6**, 28538; doi: 10.1038/srep28538 (2016).

## Supplementary Material

Supplementary Information
