# Supplementary material for: Treatment of refractory cutaneous ulcers with mixed sheets consisting of peripheral blood mononuclear cells and fibroblasts
Source: Sci Rep. 2016 Jun 22;6:28538. doi: 10.1038/srep28538 (PMC4916509; doi:10.1038/srep28538)
Supplement: Supplementary Information [file srep28538-s1.pdf]

Supporting online materials for:

Treatment of refractory cutaneous ulcers with mixed sheets consisting of peripheral blood mononuclear cells and fibroblasts

Koji Ueno, Yuriko Takeuchi, Makoto Samura, Yuya Tanaka, Tamami Nakamura, Arata Nishimoto, Tomoaki Murata, Tohru Hosoyama, Kimikazu Hamano

To whom correspondence should be addressed to:
Kimikazu Hamano (E-mail: kimikazu@yamaguchi-u.ac.jp)

This PDF file includes: Supplementary Figure S1, Supplementary Figure S2, Supplementary Figure S3.

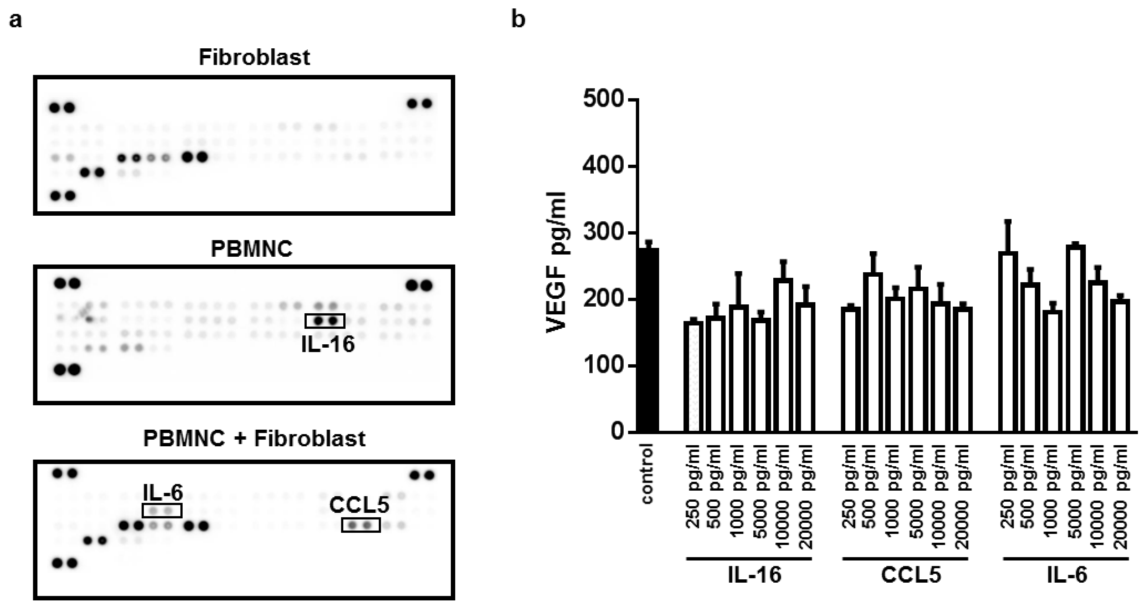

Supplementary Figure 1S.

Cytokine analysis of conditioned medium

(a) Cytokine array analysis of PBMNC-, fibroblast-, PBMNC- and fibroblast-conditioned media. (b) VEGF production in fibroblasts after the addition of IL-16, CCL5, and IL-6.

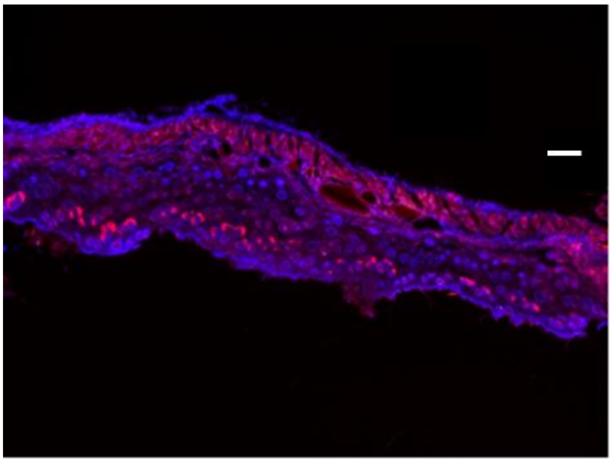

Supplementary Figure 2S.

Detection of transplanted cells and host cells 23 days after allogeneic mixed cell sheet transplantation.

GFP is a marker for transplanted cells from GFP-expressing C57BL/6 mice. H-2K^k is a marker for C3H host cells. Scale bar represents 200 μm.

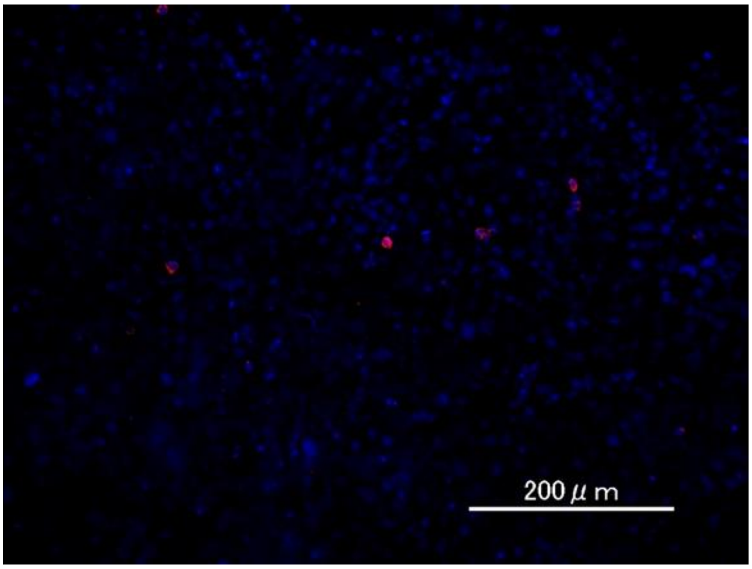

Supplementary Figure 3S.

CD11b immunostaining in mixed cell sheets.

CD11b is a marker of monocytes and macrophages and was detected in mixed cell sheets by immunostaining. The mixed cell sheets were detached form UpCell® 24 multi-well plates after PBS washing and transferred to a slide glass using a cut 1000 μl tip. The mixed cell sheets were fixed in 4% paraformaldehyde. After PBS washing, they were treated with serum-free Protein Block (Dako) and incubated with anti-CD11b antibody at room temperature for 1 h. After PBS washing, the mixed cell sheets were incubated with anti-rat IgG (H + L) secondary antibody, Alexa Fluor® 594 conjugate at room temperature for 1 h. After PBS washing, the mixed cell sheets were incubated with DAPI at room temperature for 10 min.
